# Combinatory Flowcytometric Approach in Pediatric Acute Lymphoid Leukemia Identifies Surrogate Minimal Residual Disease Markers

**DOI:** 10.3390/diagnostics15060658

**Published:** 2025-03-08

**Authors:** Noreen Grace George, Bhavika Rishi, Sanghmitra Ray, Manpreet Kaur, Raj Kamal, Shikha Garg, Sumit Mehndiratta, Nidhi Chopra, Shamsuz Zaman, Amitabh Singh, Aroonima Misra

**Affiliations:** 1 ICMR-National Institute of Child Health and Development Research (Formerly ICMR-National Institute of Pathology), New Delhi 110029, India; 2 Department of Pediatrics, Vardhman Mahavir Medical College and Safdarjung Hospital, New Delhi 110029, India; 3 Dr. Dangs Lab, New Delhi 110016, India; 4 ICMR-National Institute of Cancer Prevention, Noida 201301, India

**Keywords:** acute leukemia, flow cytometry, minimal residual disease (MRD), LAIP, DfN

## Abstract

**Background/Objectives**: Minimal residual disease (MRD) refers to the resistant clonal population of leukemia cells that survive induction chemotherapy, serving as a critical indicator of treatment response in pediatric Acute Lymphoid Leukemia (ALL). While flow cytometry (FCM) and molecular methods are standard for MRD detection, novel leukemia-associated immunophenotype (LAIP) markers are needed when conventional markers are insufficient. **Methods**: MRD was assessed in 218 pediatric B-ALL patients using a combinatory approach of Different-from-Normal (DfN) and LAIP strategies. An eight-color flow cytometry panel included routine MRD markers (e.g., CD10, CD19, and CD20) and less commonly used markers (e.g., CD123, CD73, CD86). Cytogenetic and molecular profiling were integrated to evaluate the association between genetic abnormalities and MRD positivity. **Results**: The combined DfN and LAIP approach enhanced MRD detection sensitivity compared to individual methods. CD7 showed a significant association with MRD positivity (*p* = 0.003), whereas CD73 (*p* = 0.000) and CD86 (*p* = 0.002) correlated with MRD-negative status. CD123 exhibited the highest aberrancy among MRD-positive cases, while CD81 had the lowest. These findings highlight the prognostic potential of CD73 and CD86 for MRD-negative status, complementing the established utility of CD123. **Conclusions**: Incorporating novel markers (CD123, CD73, CD86, and CD81) into MRD panels enhances detection sensitivity and clinical applicability. These markers are compatible with standard flow cytometry, supporting their integration into routine practice for comprehensive MRD evaluation, ultimately improving therapeutic outcomes in pediatric B-ALL.

## 1. Introduction

Acute lymphoblastic leukemia (ALL) is a genetically heterogeneous disease characterized by the proliferation of immature lymphocytes. B-ALL, the most common subtype in India, accounts for 85% of all cases. With rapid advancements in personalized medicine, MRD assays have surpassed conventional cytomorphologic analysis and have become the standard of care in assessing response and prognostication in ALL [1].

Accurate risk stratification using MRD evaluation requires methodologies that achieve high analytical sensitivity to detect the residual leukemic cells that are otherwise below the limits of detection by conventional cytomorphologic methods [2].

Highly sensitive molecular techniques such as reverse transcription quantitative polymerase chain reaction (RT-qPCR), next-generation sequencing (NGS), and multiparameter flow cytometry (MFC) are used to detect MRD. Although all the methods are resource-intensive, MFC is relatively economical, has fast turnaround times, and has the added advantage of detecting viable and non-viable cells [3].

The European Leukemia Net (ELN) guideline has recommended the use of multiparameter flow cytometry as a reliable technique for MRD detection [4]. This approach involves the utilization of a variety of markers, including but not limited to CD 34, CD 38, CD 45, CD 117, CD 7, CD 56, CD 13, CD 33, and CD 19. Various research studies have examined the clinical relevance of CD34, as well as CD22, CD 13, CD 33, CD 38, CD 56, CD 20, CD 10, CD 19, TDT, HLA-DR as reliable LAIP markers for MRD in childhood ALL cases characterized by favorable attributes [5,6,7]. Flow cytometry also identifies aberrant immunophenotypes in B-lymphoblasts, such as CD 58 and CD 38 antigens [8,9].

MFS MRD usually follows two approaches. First, the leukemia-associated immunophenotype (LAIP) approach involves tracking specific immunophenotypic patterns identified in leukemic blasts at diagnosis throughout the treatment course. MRD positivity in this approach is determined by detecting cells that retain these diagnostic immunophenotypic signatures post-treatment [10]. Second is the Different-from-Normal (DfN) approach, in which immunophenotypic characteristics are compared against standardized normal patterns, eliminating the requirement of initial diagnostic samples. The LAIP approach is sensitive, but does not consider immunophenotypic shifts and cannot be used when no LAIP is identified at diagnosis or when a diagnosis sample is unavailable. The DfN approach can detect these shifts, but ample expertise is needed to determine a malignant population [4].

The European LeukemiaNet (ELN) MRD Working Party recommends combining both LAIP and DfN for AML MRD monitoring [11]. This strategy, called the “LAIP-based DfN approach”, classifies leukemia-associated patterns into four broad categories: those found at diagnosis, those found during follow-up (with or without diagnostic data), and those showing new abnormalities. Although this approach is resource-intensive and has limited standardization, it has heightened sensitivity, does not need initial diagnostic samples and is applicable to over 90% of patients [5].

No similar combinatory flow cytometric guidelines or recommendations exist for B-ALL [10,11].

In this study, we used a combinatory approach involving DfN and LAIP to analyze 218 pediatric B-ALL patients. This strategy aims to expand the marker repertoire and enhance MRD sensitivity, particularly in cases negative for conventional MRD markers. The study highlights the role of MRD markers in risk stratification for relapse-prone patients, improving prognosis and tailoring chemotherapy for optimal outcomes, especially for those with intermediate or standard risk based on molecular and cytogenetic assessments.

## 2. Materials and Methods

### 2.1. Patient Inclusion and Study Population

This study retrospectively analyzed 218 pediatric patients aged 9 months to 15 years diagnosed with B-cell acute lymphoblastic leukemia (B-ALL) from 2018 to 2023 (Table 1). Diagnoses were based on morphology, cytochemistry, and immunophenotyping. The relevant records were extracted from software and case records. Ethical approvals were obtained from the respective institutional ethical committee.

### 2.2. Flow Cytometry Analysis and Gating Strategy

The FCS files of these patients were retrospectively analyzed using a combinatory gating strategy to identify surrogate markers for minimal residual disease (MRD). Bone marrow samples from 218 B-cell acute lymphoblastic leukemia (B-ALL) patients were processed according to standard protocols. Briefly, red blood cell lysis was performed using a 10×BD Pharm Lyse™ buffer (BD Biosciences, Cat# 555899, San Jose, CA, USA) prepared from a 10× stock solution, and single-cell suspensions were generated. The samples were stained with a standardized 8-color antibody panel, which included backbone markers (CD19, CD10, CD34, and CD45) and additional investigational markers relevant to MRD detection, such as CD38, CD58, CD73, CD86, CD123, CD81, and CD66c. Specific antibody clones, fluorochromes, manufacturers, catalog numbers, and compositions are detailed in Supplementary Tables S1 and S2.

For staining, approximately 2 million cells per sample were used, and the final volume was adjusted to 100 µL using sheath fluid. Post-staining, cells were acquired on a BD FACS CANTO™ II flow cytometer (BD Biosciences, San Jose, CA, USA), ensuring a minimum collection of 2.0 million events per sample. Data acquisition was executed with rigorous calibration and quality control measures.

**Figure 1:**
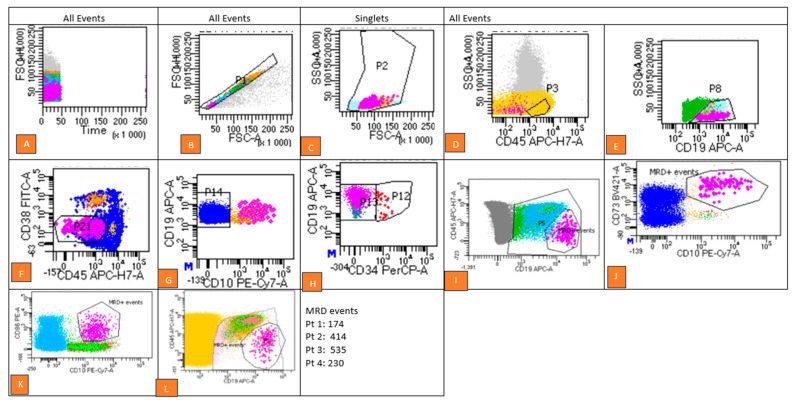
This figure outlines the gating strategy and methodology used for MRD detection, utilizing an 8-colour, 3-laser flow cytometry analysis on a BD FACS CANTO^TM^ II cytometer, with data analyzed using BD FACS DIVA ^TM^ v 8.03 software. (**A**) Time Gate vs. FSC-H: All events were initially monitored to confirm data acquisition stability. (**B**) Singlet Gate: A gate was applied on FSC-H vs. FSC-A to exclude doublets. (**C**) Viable Cell Gate: Debris, platelet clumps, and artifacts were excluded based on FSC and SSC properties to isolate viable cells. (**D**) CD45 Plot: Representative cell populations were visualized on a CD45 dot plot. (**E**) CD19+ B Cell Gate: B cells were identified using CD19 vs. SSC plots. (**F**) CD45 vs. CD19 Plot: This step refined the isolation of CD19+ cells for further analysis. (**G**) CD10 vs. CD19 Plot: Expression patterns of various markers on B cells were examined. (**H**) CD34 vs. CD19 Plot: B cell subsets, including immature (CD19+CD34+) and mature populations, were analyzed. (**I**–**L**): Residual leukemic cells (MRD) were clearly identified within predefined gates. Statistics for MRD events are provided in the figure. (Flow graphs of the patients are given in Supplementary Images).

The gating strategy comprised multiple sequential steps (Figure 1):

- Exclusion of doublets using forward scatter-area (FSC-A) versus forward scatter-height (FSC-H) plots.
- Debris removal based on forward scatter (FSC) versus side scatter (SSC) parameters.
- Separation of cell populations using CD45 versus CD19 dot plots to isolate CD19+ CD34+ (immature blasts) from CD19+ CD34− (mature B-cell populations).
- Evaluation of investigational marker expression patterns by comparing fluorescence intensities in stained samples with internal control populations. Aberrant expression patterns were identified as MRD-positive.

Data analysis was performed using BD FACS Diva™ v8.0.3 (BD Biosciences, San Jose, CA, USA) software, with MRD positivity defined as a threshold of ≥0.01% leukemic cells. High-quality dot plots with clearly demarcated gates were generated, ensuring the distinct labeling of MRD events. Gate statistics were meticulously recorded to validate the analysis’s reliability.

### 2.3. Cytogenetic and Molecular Analysis

All cases underwent conventional G-band karyotyping, according to the International System for Human Cytogenetic Nomenclature (ISCN) 2020 criteria. Molecular analysis was conducted at the time of diagnosis using commercially available kits, following the manufacturer’s instructions through RT-PCR. For molecular analysis, RNA and DNA extractions were performed using the QIamp RNA Mini Kit and QIAamp DNA Mini Kit from QIAGEN GmbH, Hilden, Germany, respectively. These were followed by RT-PCR analysis according to the manufacturer’s instructions.

### 2.4. Statistical Analysis

Statistical analysis was conducted to assess associations between categorical variables using the Chi-square test. In cases where any cell value was less than 5, the Fisher exact test was employed to ensure statistical robustness. Patient characteristics were analyzed to provide baseline characteristics of the cohort. The total number of patients included in the study was recorded as 208 out of the 218 pediatric B-ALL patients.

A *p*-value of less than 0.05 was considered statistically significant. Data analysis was performed using Stata version 15.1 software.

## 3. Results

The flow cytometry (FCM) data for all patients were analyzed and gated using a combinatory approach to identify markers for MRD employing both the DfN and LAIP approaches. A comprehensive analysis was conducted on 218 cases, comprising 38% females and 61.9% males.

### 3.1. Immunophenotypic Aberrancies

Immunophenotypic aberrancies, which refer to deviations in antigen expression on neoplastic cells compared to hematogones, can be characterized by distinct patterns. Immunophenotypic aberrancies were identified based on deviations in antigen expression (Table 2).

### 3.2. Correlation of MRD Negativity with Cytogenetic Abnormalities

Cytogenetic data were collected from all patients and analyzed for the incidence of MRD positivity in 150 out of 214 cases. These cases were categorized according to the World Health Organization (WHO) classification of hematolymphoid disorders. The major findings included hyperploidy in 26 patients and t(12;22)(p13;q22) in 13 patients, both being associated with the majority of the outcomes as MRD-negative. Other abnormalities, like t(9;22)(q34;q11) and hypoploidy, occurred less frequently. Notably, the patients with t(12;22)(p13;q22) showed favorable MRD negativity and survival outcomes (Table 3).

In addition to lymphoid antigens, aberrant myeloid antigens were frequently observed in B-ALL cases, with CD33 being the most commonly expressed antigen, followed by CD13, CD123, CD81, and CD66c (Table 4).

Table 4 compares the incidence and MRD status of various markers in the study cohort with rates reported in other studies worldwide.

Table 5 compares the incidence and MRD status of various Lymphocyte/NK lineage markers in the study cohort with rates reported in other studies. These data highlight the variability in marker prevalence and the need for individualized MRD assessment in ALL.

### 3.3. MRD Expression Data

Data from 208 patients were available. Table 6 shows the strong associations with CD7, CD73, and CD86. Notably, CD 7 demonstrated the highest MRD-positive expression rate with a *p*-value of 0.003, underscoring its potential relevance as a significant marker in differentiating MRD-positive cases. The findings emphasize the importance of CD73 and CD86, which showed strong associations with MRD-negative status, highlighting their possible utility as prognostic markers in identifying favorable outcomes or refining therapeutic strategies.

Additionally, CD 73 demonstrated a higher preference for MRD-negative samples, which is noteworthy and warrants further investigation. The strong statistical association suggests that incorporating these markers into the current diagnostic flow cytometry panels may enhance the identification of MRD, especially in cases where the conventional LAIP method fails.

### 3.4. Risk Stratification

Risk stratification of the cohort showed that that 69% of patients were classified as high risk (HR), 14.7% as intermediate risk (IR), and 16.3% as standard risk (SR). Table 7 details the MRD-positive samples’ risk stratification and current status.

## 4. Discussion

This study retrospectively analyzed flow cytometric and clinical data of 218 patients with B-ALL to investigate the utility of novel markers for MRD detection and their association with patient outcomes. The integration of LAIP and DfN approaches demonstrated superior marker detection compared to either method alone.

This combinatorial strategy enabled the identification of additional aberrant markers, including CD58, CD123, CD3, and CD7, at frequencies that matched or exceeded those reported in previous studies [16,17]. Notably, CD58 was detected in 19% of cases, surpassing the typical 10–20% reported in the literature, while CD123 was observed in 12% of cases, slightly higher than the conventional 5–10% incidence. The enhanced detection rates can be attributed to our methodology’s improved sensitivity in identifying low-density or dimly expressed antigens that standard approaches might overlook.

The study also revealed abnormal expression patterns across multiple markers including HLA-DR, CD22, CD33, CD58, CD123, CD13, CD73, CD86, CD81, CD66c, CD7, CD3, and CD2. The integration of LAIP and DfN approaches proved particularly effective, enabling detection rates that exceeded conventional methods. Notably, CD58 was detected in 19% of cases, surpassing the typical 10–15% reported in the literature, while CD123 was found in 12% of cases, compared to the usual 5–10% incidence [25]. CD3 and CD7 showed frequencies of 2.4% and 4.8%, respectively, slightly above previously reported rates of 1–3% [26,28]. A particularly significant finding was the strong association between CD73 expression and MRD-negative status. This correlation suggests CD73’s potential utility as a prognostic marker and its possible role in identifying favorable outcomes. Similarly, CD86 demonstrated promising results as an adjunct marker for MRD detection, with both CD73 and CD86 showing statistical significance (*p* < 0.05). These findings align with a recent study by Słota et al., which reported increased CD73 expression during early treatment phases [19].

The combined LAIP and DfN approach offered several methodological advantages too, including enhanced sensitivity in detecting low-density antigens, improved identification of subtle immunophenotypic aberrancies, and more comprehensive marker detection compared to single-method approaches. These benefits translate to significant clinical implications, particularly in the need for broader marker panels in initial diagnosis and MRD monitoring, the potential for improved risk stratification through novel marker integration, and enhanced ability to identify residual disease in cases where conventional markers prove insufficient.

Similar to earlier studies, our findings also support more nuanced treatment approaches, particularly in high-risk cases requiring aggressive treatment protocols [29]. The optimal timing for MRD evaluation was identified between days 35 and 42 post-induction, with marker compatibility with standard fluorochromes (FITC, PE, APC) facilitating relatively easier clinical integration [8,27,30]. However, several limitations warrant consideration, including sample size constraints for certain marker subgroups and potential population-based variability in marker expression. Future research should focus on validating CD123’s role in larger cohorts, developing standardized MRD assessment protocols, and investigating marker utility in MRD-negative cases prone to relapse.

The study also revealed important insights regarding myeloid antigen expression in B-ALL. We observed higher frequencies of myeloid markers compared to previous reports, with CD33 being the most prevalent, followed by CD13, CD123, CD81, and CD66c. The co-expression of CD13 and CD33 emerged as the most common aberrancy pattern, highlighting the complex immunophenotypic landscape of B-ALL. Compared to other published studies, which reported variable frequencies of aberrant myeloid antigen expression ranging from 10% to 40%, our findings demonstrate a higher prevalence, particularly of CD33, CD13, and CD11b. Expression of CD123 and CD66c was also noted in specific cases (Pt6, Pt8, Pt9), highlighting the diversity of myeloid antigen expression patterns in B-ALL.

Surrogate epigenetic markers, such as DNA methylation patterns and histone modifications, could also serve as potential MRD markers, especially in cases where traditional genetic markers are uninformative [25,31,32]. Patients with favorable cytogenetic features but persistent MRD may receive more aggressive therapy than traditionally indicated based solely on genetics. Conversely, patients with high-risk genetic features who achieve rapid MRD negativity may be spared overly intensive therapy, reducing treatment-related toxicity without compromising outcomes. Our cytogenetic analysis also yielded interesting observations regarding the relationship between genetic abnormalities and MRD status. Notably, 84% of patients with hyperdiploidy, traditionally associated with a good prognosis, showed MRD positivity. This unexpected finding suggests that conventional risk stratification methods may need refinement, possibly through the integration of additional genetic markers or mutation analysis [33]. Although 40% of patients in our cohort with hyperploidy had a good prognosis, 84% of them turned out to be MRD-positive. This signifies that the patient risk stratification at diagnosis needs additional categorization like certain mutations or genetic characteristics that might have been missed.

More prognostic variables are required for optimized risk stratification so that MRD positivity is reduced because the first attempt at treatment with a standard risk-stratified chemotherapy regime is the best possible chance for a cure in ALL. Relapses often do not respond to second line of chemotherapy. Relapse was more common in the cases of good and intermediate prognostic patients as compared to that of poor prognosis because the chemotherapy was ineffective and certain prognostic factors remained undiagnosed at baseline because of a lack of next-generation sequencing.

The focus is on the detection of specific fusion genes (e.g., t(9;22)(q34;q11), TEL-AML1), gene rearrangements, or mutations (e.g., NPM1, FLT3) that are unique to the leukemic clone [34]. This specificity allows for precise monitoring of MRD and the detection of relapse even before clinical symptoms appear. This patient cohort had 28 MRD-positive patients with molecular and cytogenetic abnormalities.

t(12;22)(p13; q22) is the most common translocation found in childhood ALL, and in this patient cohort, the incidence of t(12;22)(p13;q22) was also higher. TEL-AML1 (ETV6-RUNX1) is regarded as an early genetic hit that develops a pre-leukemia clone during pregnancy. Patients with t(12;22)(p13;q22) fusion tend to respond well to treatment, and the presence of this fusion is associated with a lower risk of relapse. Achieving early MRD negativity in these patients is particularly predictive of a favorable prognosis.

It is subsequently succeeded by the presence of t(9;22)(q34;q11) in one patient out of the eleven, who tested positive for MRD and subsequently succumbed during the maintenance phase. MRD levels in positive t(9;22)(q34;q11) patients are critical for assessing disease progression and relapse risk [35]. High MRD levels in these patients are often associated with a higher likelihood of relapse and poorer overall survival. Early and sustained MRD negativity is crucial for improving outcomes in t(9;22)(q34;q11) positive ALL, and targeted therapies such as tyrosine kinase inhibitors (TKIs) are essential for managing these patients [32,33]. The occurrence of the chromosomal translocation (1;19)(q23; p13.3)/TCF3(E2A)-PBX1 is observed in a small percentage of adult ALL cases and a slightly larger percentage of pediatric ALL cases [36]; in this cohort, we observed eight patients with this translocation and one patient was MRD-positive and relapsed.

Patients exhibiting hypoploidy tested negative for MRD, while those with hyperploidy had an MRD positivity rate of 12.5%, with the remaining cases testing negative for MRD. High hyperdiploidy is an uncommon cytogenetic subtype of childhood ALL characterized by karyotypes displaying an atypical modal chromosome count ranging from 51 to 67 chromosomes. This subset constitutes a substantial proportion, comprising 25–30% of patients with B-cell ALL. In the present study, a total of 12 pediatric patients were identified as having this specific cytogenetic anomaly.

The presence of specific translocations showed varying associations with MRD status and outcomes. Patients with t(12;22)(p13;q22) generally demonstrated favorable responses, while the single case of t(9;22)(q34;q11) with MRD positivity had a poor outcome. These findings reinforce the importance of considering both genetic and immunophenotypic markers in risk assessment.

Several limitations of our study warrant consideration. The relatively small sample size for certain marker subgroups may limit the generalizability of some findings. Additionally, marker expression variability across different populations needs further investigation. Future research should focus on validating these findings in larger, prospective cohorts and exploring the potential of these novel markers in combination with emerging therapies.

The clinical implications of our findings are significant. The integration of markers like CD73 and CD86 into routine MRD assessment panels could enhance detection sensitivity, particularly in cases where conventional markers prove insufficient. Furthermore, the observed patterns of myeloid antigen expression suggest the need for broader marker panels in initial diagnosis and MRD monitoring.

Looking ahead, several areas deserve further investigation. The role of CD123 as an exploratory marker requires validation in larger cohorts, particularly given its potential significance in MRD-negative cases prone to relapse. Additionally, the development of standardized protocols incorporating both conventional and novel markers would be valuable for harmonizing clinical practice.

## 5. Conclusions

Our combinatory approach, integrating the LAIP and DfN techniques, demonstrated enhanced specificity for marker detection compared to either technique alone. Specifically, CD123 and CD3 showed overexpression, while showing CD81 underexpression in B-ALL. Notably, CD73 displayed a strong association with MRD-negative status in CD73-positive samples, indicating its potential as a valuable marker in MRD studies. Markers such as CD123, CD86, CD73 and CD81 can complement routine MRD estimation panels, particularly when conventional markers fail to provide sufficient information.

## Figures and Tables

**Table 1:** Clinical description of patients (*n* = 218).

Parameter	Total (*n* (%))
Total Patients	218
Age Range	9 months–15 years
Median	7.5 years (MRD-positive) 6.5 years (MRD-negative)
Gender	Male 130 (59.6%), Female 88 (40.4%)
No. of Patients under different risks	High Risk 130 (59.6%), Intermediate Risk 58 (26.6%), Standard Risk 30 (13.8%)
No. of patients with different cytogenetic abnormalities	Hyper-ploidy 26 (11.9%), Hypoploidy 4 (1.8%), Others 20 (9.2%)
Treatment Response (MRD)	Positive 80 (36.7%), Negative 138 (63.3%)

**Table 2:** Aberrant Expression of flowcytometric surface antigens in B-Acute Lymphoblastic leukemia patients that were MRD-positive at the end of induction based on LAIP method and DfN combinatory approach.

Markers	Incidence		MRD Positive		Incidence Cited by Other Studies Refs %
	No. of: Positive	Positive %	No. of Cases Out of 208	Positive %	
CD 10	190	93.6	35	83	~70–83 [12,13]
CD 19	194	95.6	33	79	~90–95 [12,13]
CD 79a	127	62.6	17	40	~40–50 [14]
HLA-DR	180	88.7	35	83	~15–25 [15]
CD 58	19	9.4	8	19	~10–20 [16,17]
TdT	18	8.9	5	12	~20–70 [14]
CD 38	88	43.3	17	40	~20–40 [18]
CD 34	142	70.0	25	60	~50–70 [11]
CD 22	37	18.2	6	14	~10–20 [7]
CD 73	10	4.9	8	19	<5 [19]
CD 86	9	4.4	7	17	~50 [20]

**Table 3:** Cytogenetic profile.

Recurrent Genetic Abnormality	Prognosis	Total 65 Patients	MRD-Positive	MRD-Negative	Alive	Expired	Relapse
Hyper ploidy: >50 chromosomes	Good	26	22	4	20	3	3
Hypoploidy: <45 chromosomes	Poor	4	1	3	3	1	0
t (9;22)(q34;q11)	Intermediate	12	1	11	10	2	0
t (12;22)(p13;q22)	Good	13	1	12	10	2	1
t (1;19)(q23;p13)	Intermediate	10	3	7	7	1	1

**Table 4:** Myeloid Antigen Expression in B Acute Lymphoblastic Leukemia patients that were MRD-positive.

Markers	Incidence		MRD-Positive		Incidence Cited by Other Studies Refs %
	No. of Positive	Positive %	No. of Cases Out of 208	Positive %	
CD 13	14	6.9	2	5	~30–50 [4,16]
CD33	20	9.9	5	12	~10–25 [4,16]
CD 11b	3	1.5	2	5	>1 [21]
CD 66c	5	2.5	1	2	~40 [14,22]
CD 14	2	1.0	1	2	~10 [13]
MPO	1	0.5	0	0	~2 [23]
CD 81	6	3.0	1	2	~90–95 [24]
CD 123	14	6.9	5	12	~8.2 [25]

**Table 5:** Lymphocyte/NK lineage flowcytometric markers expressed in B Acute Lymphoblastic Leukemia patients that were MRD-positive.

Markers	Incidence		MRD Status		Incidence Cited by Other Studies Refs %
	No. of Positive	Positive %	No. of Cases Out 208	Positive	
CD 2	2	1	1	2.4	~3.6 [11]
CD 3	2	1	1	2.4	~25 [14,15]
CyCD 3	2	1	1	2.4	~5 [26,27]
SurCD 3	4	2	1	2.4	~20 [26]
CD 5	2	1	0	0.0	~3 [11]
CD 7	4	2	2	4.8	~20 [14,15]
CD 8	2	1	0	0.0	~3 [11]
CD 56	3	1	1	2.4	~4.3–6 [26]

**Table 6:** Association between positive CD expression and MRD status.

CD Markers	Total ((*n*) = 208)	MRD-Positive (%)	MRD-Negative (%)	*p*-Value
CD10	186	83.3	16.7	0.109
CD20	81	85.2	14.8	0.365
CD22	36	86.1	13.9	0.501
CD7	122	88.5	11.5	0.003 *
CD73	9	22.2	77.8	0.00 *
CD86	7	28.6	71.4	0.002 *
CD64	2	100	0	1.0
CD2	1	0	100	0.178
CD3	1	0	100	0.178
CyCD3	1	0	100	0.178
CD5	1	100	0	1.0
CD8	1	100	0	1.0
CD 56	1	100	0	1.0
CD 38	85	17.7	82.35	0.970
CD 34	136	15.4	84.6	0.228
HLA-DR	175	17.7	82.3	1.0
TdT	18	22.2	11.8	0.533
CD13	13	7.7	92.3	0.471
CD 33	19	21.1	82.6	0.752
CD 14	1	100	0	0.178
CD 11b	1	100	0	0.178
CD 81	5	20	8	1.0
CD 58	15	33.3	66.7	0.150
CD 66c	3	0	100	1.0
CD 123	13	30.8	69.2	0.253

* Significant if *p*-value <0.05.

**Table 7:** MRD-positive samples risk stratification and current status.

Risk Status	Total no.: 184	MRD-Positive	MRD-Positive			
			Expired	Alive	Survivors	Defaulter
HR	127	24	7	13	2	1
IR	27	2	0	2	0	0
SR	30	3	1	2	0	0

## Data Availability

The data presented in this study are available on request from the corresponding author. The data are not publicly available due to privacy and ethical concerns.

## Supplementary Materials

The following supporting information can be downloaded at: https://www.mdpi.com/article/10.3390/diagnostics15060658/s1, Figure S1: Flowcytometry images Patient 1–Patient 4; Table S1: Flowcytometry reagents; Table S2: Antibody panel composition.
