# Supplementary material for: Combinatory Flowcytometric Approach in Pediatric Acute Lymphoid Leukemia Identifies Surrogate Minimal Residual Disease Markers
Source: Diagnostics (Basel). 2025 Mar 8;15(6):658. doi: 10.3390/diagnostics15060658 (PMC11941652; doi:10.3390/diagnostics15060658)
Supplement: Supplementary file 1 [file diagnostics-15-00658-s001.zip › Figure S1.pdf]

Patient 1

BD FACSDiva 8.0.3

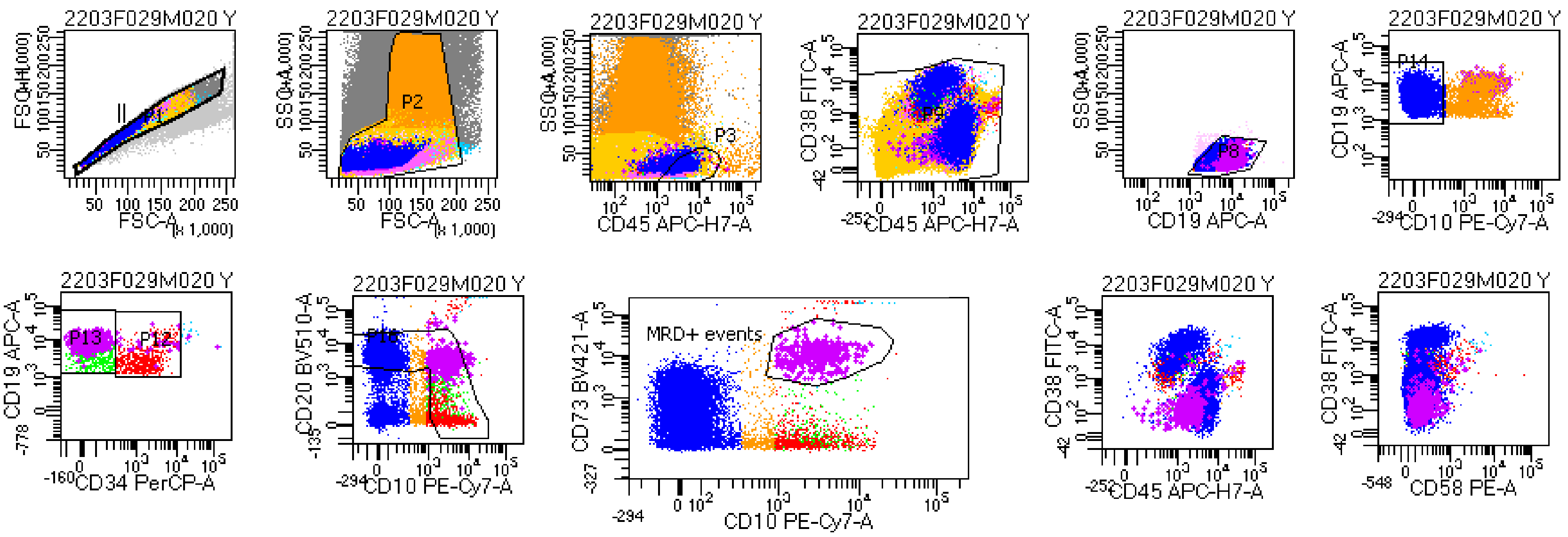

Tube: MRD2

Population	#Events	%Parent	%Total
All Events	1,724,080	####	100.0
P1	1,697,879	98.5	98.5
P3	228,059	13.4	13.2
P2	1,534,550	90.4	89.0
P4	657,926	42.9	38.2
P9	657,909	100.0	38.2
P5	21,840	3.3	1.3
P6	21,441	98.2	1.2
P7	13,891	64.8	0.8
P8	13,562	97.6	0.8
P10	1,589	11.7	0.1
P12	742	46.7	0.0
P13	820	51.6	0.0
P16	1,352	85.1	0.1
MRD+ events	414	26.1	0.0
P14	11,517	84.9	0.7

Patient 1

BD FACSDiva 8.0.3

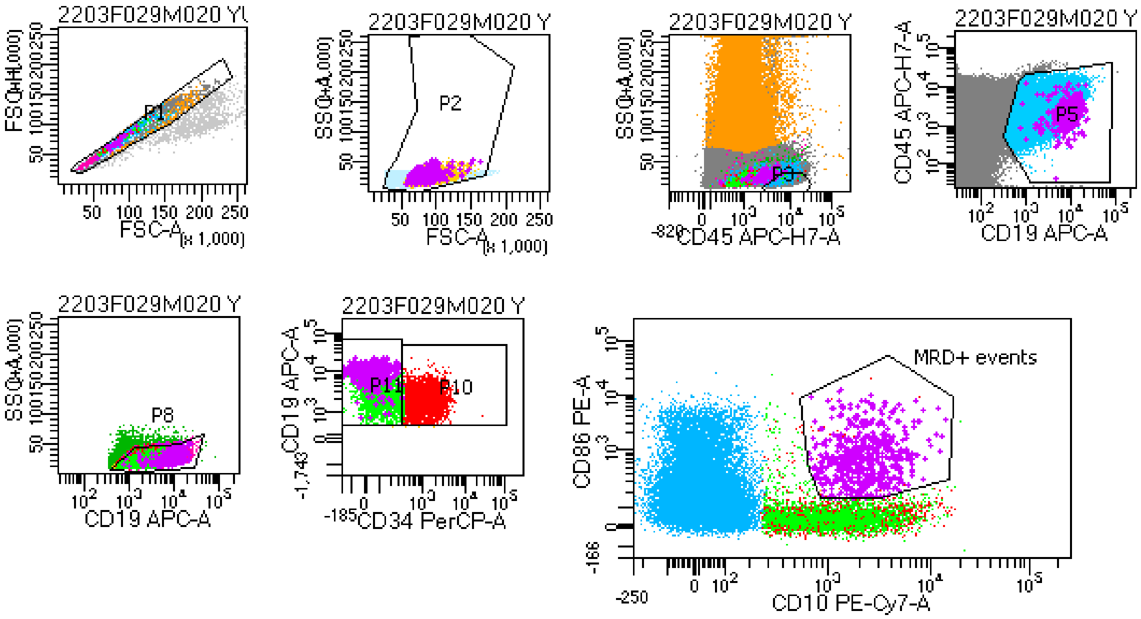

Tube: MRD3

Population	#Events	%Parent	%Total
All Events	1,931,770	####	100.0
P1	1,898,080	98.3	98.3
P3	195,370	10.3	10.1
P2	1,837,892	96.8	95.1
P4	542,863	29.5	28.1
P5	30,150	5.6	1.6
P6	29,223	96.9	1.5
P7	25,423	87.0	1.3
P8	24,156	95.0	1.3
P9	5,243	21.7	0.3
P10	2,489	47.5	0.1
P11	2,724	52.0	0.1
MRD+ events	535	19.6	0.0
P14	18,326	75.9	0.9

Patient 2

BD FACSDiva 8.0.3

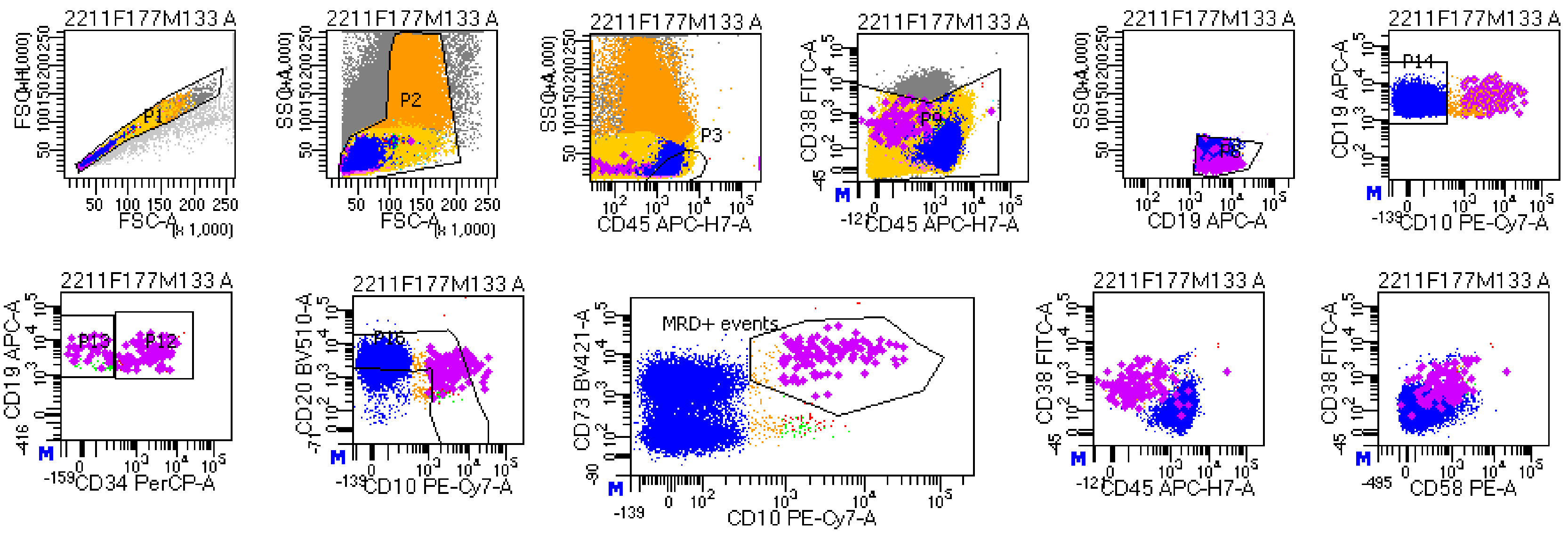

Tube: MRD2

Population	#Events	%Parent	%Total
All Events	810,037	####	100.0
P1	796,046	98.3	98.3
P3	107,410	13.5	13.3
P2	742,367	93.3	91.6
P4	428,798	57.8	52.9
P9	422,831	98.6	52.2
P5	19,512	4.6	2.4
P6	19,439	99.6	2.4
P7	18,245	93.9	2.3
P8	18,211	99.8	2.2
P10	208	1.1	0.0
P12	127	61.1	0.0
P13	81	38.9	0.0
P16	161	77.4	0.0
MRD+ events	120	57.7	0.0
P14	17,862	98.1	2.2

Patient 2

BD FACSDiva 8.0.3

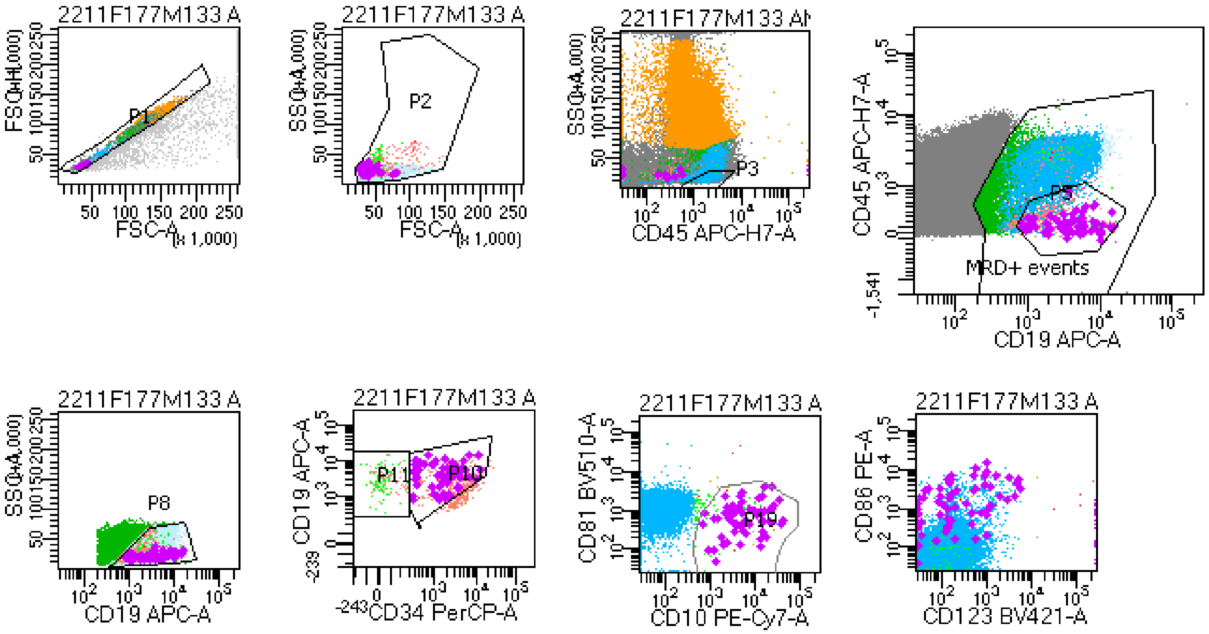

Tube: MRD3

Population	#Events	%Parent	%Total
All Events	698,740	####	100.0
P1	647,349	92.6	92.6
P3	61,324	9.5	8.8
P2	600,486	92.8	85.9
P4	303,006	50.5	43.4
P5	25,779	8.5	3.7
P6	25,758	99.9	3.7
P7	25,669	99.7	3.7
P8	21,154	82.4	3.0
P9	674	3.2	0.1
P12	518	76.9	0.1
NOT(P12)	156	23.1	0.0
P10	70	44.9	0.0
P19	66	94.3	0.0
MRD+ events	63	95.5	0.0
P11	76	48.7	0.0
P14	20,369	96.3	2.9
P24	666,142	95.3	95.3

Patient 3

BD FACSDiva 8.0.3

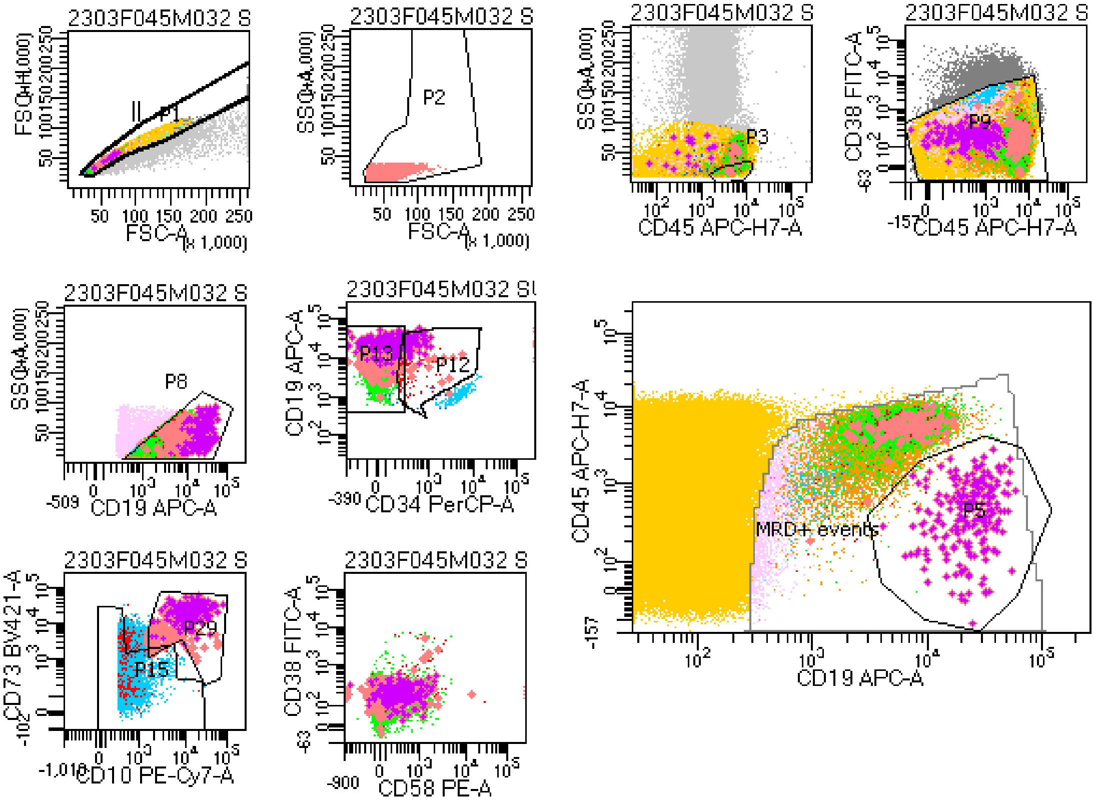

Tube: MRD2

Population	#Events	%Parent	%Total
All Events	802,056	####	100.0
P1	758,345	94.6	94.6
P3	159,437	21.0	19.9
P2	704,839	92.9	87.9
P4	504,475	71.6	62.9
P9	490,373	97.2	61.1
P5	55,355	11.3	6.9
P6	55,260	99.8	6.9
P7	55,050	99.6	6.9
P8	52,599	95.5	6.6
P10	10,167	19.3	1.3
P13	9,808	96.5	1.2
P15	6,715	66.0	0.8
P12	353	3.5	0.0
P29	351	3.5	0.0
MRD+ events	230	65.5	0.0
P11	308	0.6	0.0

Patient 3

BD FACSDiva 8.0.3

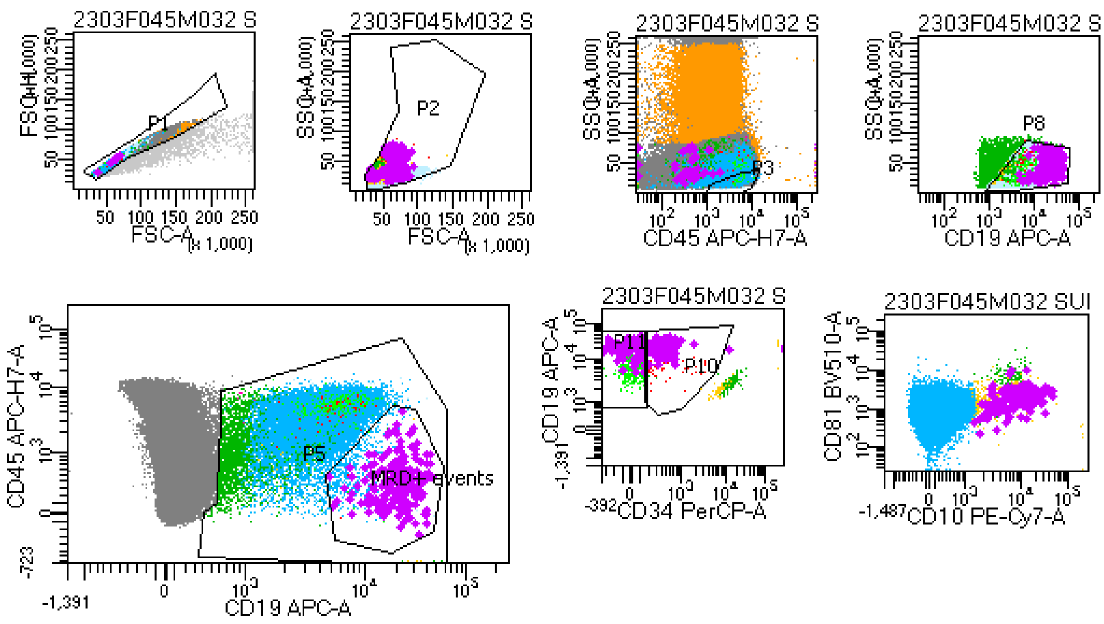

Tube: MRD3

Population	#Events	%Parent	%Total
All Events	723,304	####	100.0
P1	635,153	87.8	87.8
P3	119,825	18.9	16.6
P2	538,791	84.8	74.5
P4	390,080	72.4	53.9
P5	43,871	11.2	6.1
P6	43,828	99.9	6.1
P7	43,781	99.9	6.1
P8	39,720	90.7	5.5
P9	715	1.8	0.1
P11	369	51.6	0.1
P13	349	48.8	0.0
P15	494	69.1	0.1
P16	550	76.9	0.1
P17	453	63.4	0.1
P18	485	67.8	0.1
P10	145	20.3	0.0
P12	281	39.3	0.0
MRD+ events	174	61.9	0.0
P14	38,917	98.0	5.4

Patient 4

BD FACSDiva 8.0.3

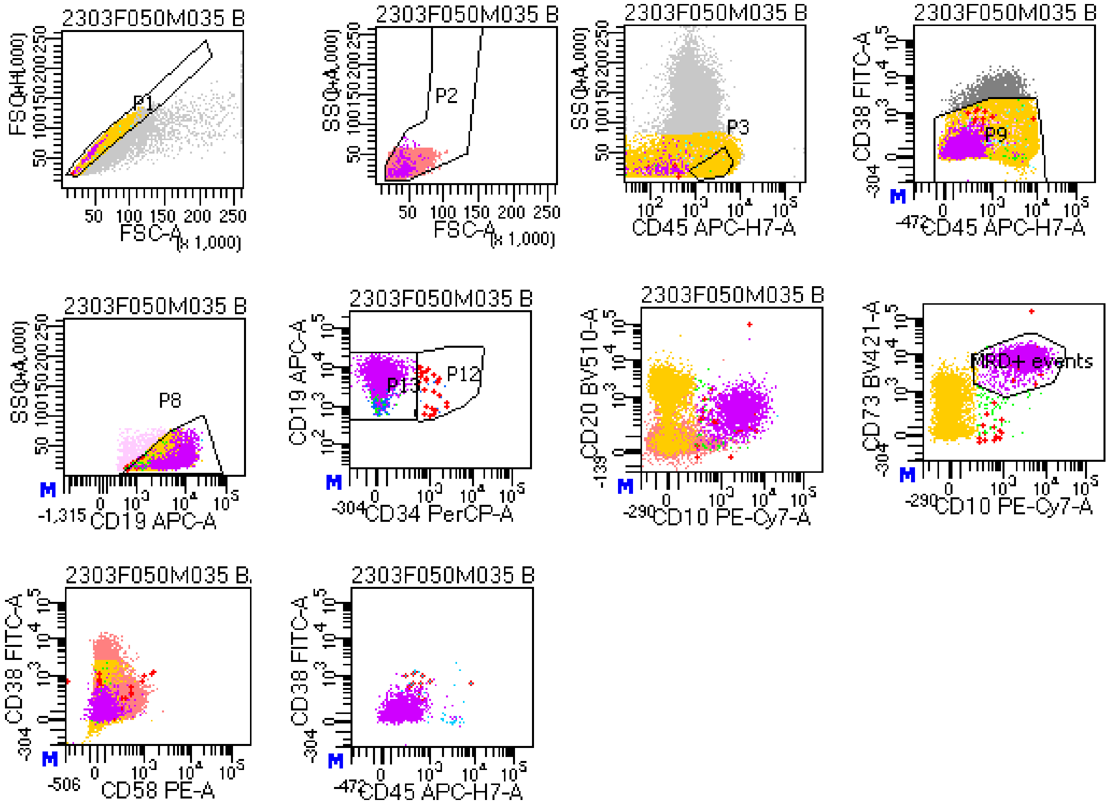

Tube: MRD 2

Population	#Events	%Parent	%Total
All Events	901,784	####	100.0
P1	820,578	91.0	91.0
P3	108,673	13.2	12.1
P2	660,878	80.5	73.3
P4	552,977	83.7	61.3
P9	551,442	99.7	61.2
P5	7,878	1.4	0.9
P6	7,663	97.3	0.8
P7	7,585	99.0	0.8
P8	6,769	89.2	0.8
P10	1,939	28.6	0.2
P13	1,912	98.6	0.2
MRD+ events	1,839	96.2	0.2
P12	26	1.3	0.0
P11	615	9.1	0.1
P22	4,835	71.4	0.5

Patient 4

BD FACSDiva 8.0.3

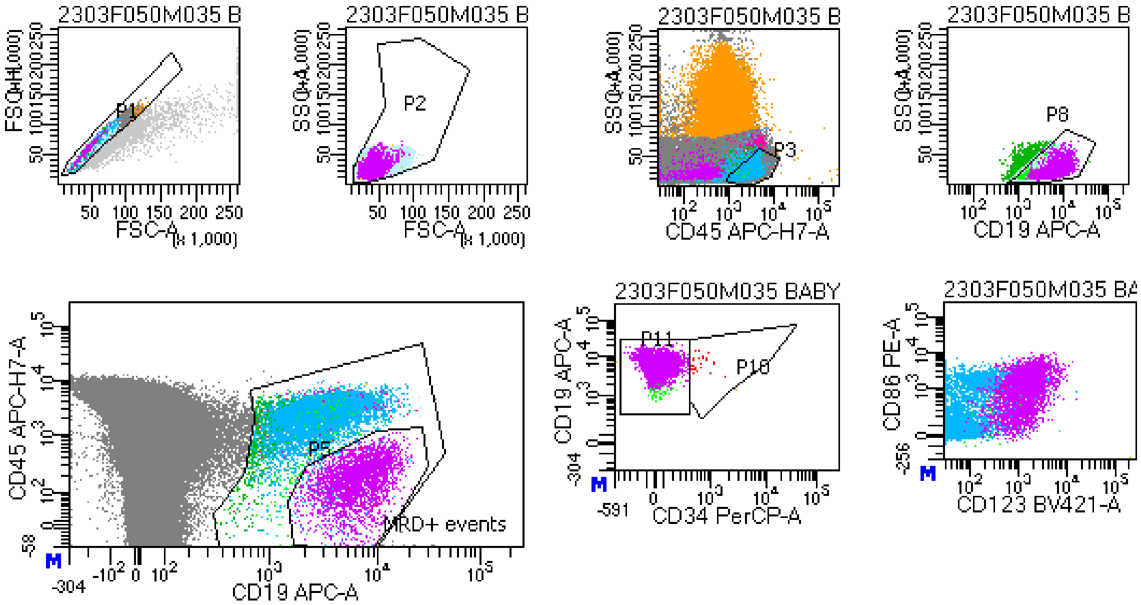

Tube: MRD 3

Population	#Events	%Parent	%Total
All Events	1,062,853	####	100.0
P1	1,001,260	94.2	94.2
P3	157,914	15.8	14.9
P2	951,250	95.0	89.5
P4	769,528	80.9	72.4
P5	15,018	2.0	1.4
P6	14,548	96.9	1.4
P7	12,837	88.2	1.2
P8	11,611	90.4	1.1
P9	3,047	26.2	0.3
P11	3,019	99.1	0.3
MRD+ events	2,901	96.1	0.3
P10	29	1.0	0.0
P14	8,519	73.4	0.8
P24	1,056,781	99.4	99.4
