# Supplementary material for: Combinatory Flowcytometric Approach in Pediatric Acute Lymphoid Leukemia Identifies Surrogate Minimal Residual Disease Markers
Source: Diagnostics (Basel). 2025 Mar 8;15(6):658. doi: 10.3390/diagnostics15060658 (PMC11941652; doi:10.3390/diagnostics15060658)
Supplement: Supplementary file 1 [file diagnostics-15-00658-s001.zip › Supplementary tables.pdf]

Supplementary Table S1: Flowcytometry reagents

Sl.No.	Brand names	Dye	Manufacturers	Catalogue numbers	clone
1	CD10	PE-cy7	BD Bioscience	341092	HI10a
2	CD34	PerCP	BD Bioscience	340430	8G12
3	CD19	APC	BD Bioscience	340437	SJ25C1
4	CD45	APC-H7	BD Bioscience	641399	2D1
5	CD38	FITC	BD Bioscience	340927	HB7
6	CD58	PE	BD Bioscience	340295	L306.4
7	CD73	BV421	BD Bioscience	562430	AD2
8	CD20	BV510	BD Bioscience	563067	2H7
9	CD86	PE	BD Bioscience	555658	2331 (FUN-1)
10	CD123	BV421	BD Bioscience	562517	9F5
11	CD81	BV510	BD Bioscience	740177	JS-81
12	Mouse IgG1	FITC	BD Bioscience	349041	X40
13	Mouse IgG1	PE	BD Bioscience	349043	X40
14	Mouse IgG1	BV421	BD Bioscience	562438	X40
15	PharmLysis Buffer 10x	-	BD Bioscience	555899	-
16	Sheath	-	BD Bioscience	342003	-
17	FACS Tubes	-	BD Bioscience	352054	-

❖ Supplementary Table S2: Antibody panel composition

Sl.no	Fluorochrome	CD Marker	Purpose
1	PE-cy7	CD10	Backbone Marker
2	PerCP	CD34	Backbone Marker
3	APC	CD19	Backbone Marker
4	APC-H7	CD45	Backbone Marker
5	FITC	CD38	LAIP
6	PE	CD58	LAIP
7	BV421	CD73	LAIP

8	BV510	CD20	LAIP
9	PE	CD86	LAIP
10	BV421	CD123	LAIP
11	BV510	CD81	LAIP
12	FITC	Mouse IgG1	To check non specific Binding
13	PE	Mouse IgG1	To check non specific Binding
14	BV421	Mouse IgG1	To check non specific Binding
